# A novel lncRNA LOC101928222 promotes colorectal cancer angiogenesis by stabilizing HMGCS2 mRNA and increasing cholesterol synthesis

**DOI:** 10.1186/s13046-024-03095-8

**Published:** 2024-07-04

**Authors:** Lisha Chang, Jie Ding, Juan Pu, Jing Zhu, Xiang Zhou, Qian Luo, Jie Li, Mengsen Qian, Shuhui Lin, Juan Li, Keming Wang

**Affiliations:** 1 Department of Oncology, The Second Affiliated Hospital of Nanjing Medical University, Nanjing, Jiangsu China; 2 Department of Oncology, Lianshui County People’s Hospital, Affiliated Hospital of Kangda college, Nanjing Medical University, Huaian, Jiangsu China; 3 Head and neck surgery, The Second Affiliated Hospital of Nanchang University, Nanchang, Jiangxi China

**Keywords:** Colorectal cancer, LOC101928222, Angiogenesis, m6A, Cholesterol synthesis

## Abstract

**Background:**

Metastasis is the leading cause of mortality in patients with colorectal cancer (CRC) and angiogenesis is a crucial factor in tumor invasion and metastasis. Long noncoding RNAs (lncRNAs) play regulatory functions in various biological processes in tumor cells, however, the roles of lncRNAs in CRC-associated angiogenesis remain to be elucidated in CRC, as do the underlying mechanisms.

**Methods:**

We used bioinformatics to screen differentially expressed lncRNAs from TCGA database. LOC101928222 expression was assessed by qRT-PCR. The impact of LOC101928222 in CRC tumor development was assessed both in vitro and in vivo. The regulatory mechanisms of LOC101928222 in CRC were investigated by cellular fractionation, RNA-sequencing, mass spectrometric, RNA pull-down, RNA immunoprecipitation, RNA stability, and gene-specific m6A assays.

**Results:**

LOC101928222 expression was upregulated in CRC and was correlated with a worse outcome. Moreover, LOC101928222 was shown to promote migration, invasion, and angiogenesis in CRC. Mechanistically, LOC101928222 synergized with IGF2BP1 to stabilize HMGCS2 mRNA through an m6A-dependent pathway, leading to increased cholesterol synthesis and, ultimately, the promotion of CRC development.

**Conclusions:**

In summary, these findings demonstrate a novel, LOC101928222-based mechanism involved in the regulation of cholesterol synthesis and the metastatic potential of CRC. The LOC101928222-HMGCS2-cholesterol synthesis pathway may be an effective target for diagnosing and managing CRC metastasis.

**Supplementary Information:**

The online version contains supplementary material available at 10.1186/s13046-024-03095-8.

## Background

Colorectal cancer (CRC) is a commonly occurring malignancy that affects the gastrointestinal system. Globally, CRC is associated with the third highest rates of morbidity and mortality of all malignant tumors. More than 60% of patients with CRC have metastasis at the time of first diagnosis and the mean 5-year survival rate for CRC is approximately 60% [1, 2]. Massive angiogenesis underlies tumor growth and metastasis. Tumor blood vessels play a crucial role in promoting tumor development and invasion by facilitating the transportation of essential nutrients and metabolites. In addition, these blood vessels serve as conduits for the metastasis of tumor cells [3]. While many anti-angiogenic drugs are used clinically, the complexity of the angiogenic regulatory network has limited their therapeutic benefit [4]. Therefore, identifying novel mechanisms associated with CRC angiogenesis may provide meaningful perspectives and effective approaches for identifying and managing CRC.

Studies have shown that many aberrantly expressed protein-coding genes are involved in CRC. However, protein-coding genes account for less than 2% of the human genome, and more than 98% of the genome is transcribed into noncoding RNAs [5]. Long noncoding RNAs (lncRNAs) are RNA molecules with lengths that exceed 200 nucleotides and do not encode functional proteins [6]. Growing evidence indicates that lncRNAs play a significant role in several biological processes within tumor cells, including stemness, metastasis, metabolism, immune evasion, and angiogenesis [7–9]. LncRNAs interact with DNA, RNA, and proteins, regulating chromatin structure and function, along with the transcription of genes, and influence RNA splicing, stability, and translation [10, 11]. However, the possible activities and precise roles of lncRNAs in CRC remain incompletely understood.

Cholesterol is an essential lipid component of mammalian cell membranes and acts as a precursor for the biosynthesis of bile acids and steroid hormones, which play a key role in cell signaling [12, 13]. Abnormal lipid metabolism is commonly observed in tumors. Recent studies have indicated that abnormal cholesterol metabolism is correlated with all stages of cancer, and can promote rapid proliferation and metastasis, immune evasion, and autophagy dysfunction in tumors [14, 15]. Tumor incidence and/or mortality are correlated with blood cholesterol levels [16]. Previous work has demonstrated that HGF induces cholesterol biosynthesis by activating the c-Met/mTOR pathway, leading to CRC liver metastasis [17]. However, the precise involvement of cholesterol in CRC angiogenesis is poorly understood.

In this study, we identified a hitherto uncharacterized lncRNA, LOC101928222. Our findings indicated that LOC101928222 is significantly upregulated in CRC and exerts a pivotal influence on the invasive, metastatic, and angiogenic potential of CRC. We further found that LOC101928222 can interact with IGF2BP1 and enhance the stability of HMGCS2 mRNA in an m6A-dependent manner, thereby promoting cholesterol synthesis, and finally leading to the malignant CRC phenotype.

## Methods

### Bioinformatics analysis

The expression patterns obtained using RNA-sequencing at level 3, together with the corresponding clinical data for CRC, were obtained from the TCGA dataset, accessible at (https://portal.gdc.com). GTEx datasets, specifically the V8 release, were acquired via the official GTEx data portal (https://www.gtexportal.org/home/datasets). Statistical analyses were performed using R software version 4.0.3 (R Foundation for Statistical Computing, Vienna, Austria). A *p*-value < 0.05 was considered statistically significant [18].

### Clinical specimens

CRC and paracancerous tissues were collected from surgical patients at The Second Affiliated Hospital of Nanjing Medical University. None of the patients received radiotherapy or chemotherapy before surgery. Tissue specimens were immediately cryopreserved in liquid nitrogen following surgical excision. All participants provided written informed consent before their involvement in the study. Ethical approval for the study was provided by the Medical Ethics Committee of Nanjing Medical University (approval no: [2019]-KY-121).

### Cell culture and transfection

Six CRC cell lines (SW480, SW620, HT29, HCT-116, LOVO, and DLD-1), normal intestinal epithelial fetal human colon (FHC) cells, and human umbilical vein endothelial cells (HUVECs) were procured from the Cell Bank of the Chinese Academy of Sciences and the American Type Culture Collection, respectively. The cells were grown in DMEM or RPMI1640 (Gibco, Grand Island, NY, USA) supplemented with 10% fetal bovine serum (FBS) and 1% penicillin/streptomycin. Cultures were maintained in an incubator at 37 °C with 5% CO_2_ [19].

ShRNAs targeting LOC101928222, the HMGCS2 and LOC101928222 overexpression plasmid, and other targeting shRNAs were designed by and obtained from GenePharma Company (Shanghai, China). The shRNAs were transfected into CRC cells using Lipofectamine 2000 (Invitrogen, Carlsbad, CA, USA) following the manufacturer’s guidelines [20]. The sequences of the shRNAs are shown in Table S1.

### RNA extraction and qRT-PCR

Total RNA was isolated from tissues and cells using Trizol reagent (Invitrogen, Carlsbad, CA, USA) and reverse-transcribed into cDNA. qPCR was performed with SYBR Green Master Mix (Vazyme, Nanjing, China) according to the manufacturer’s instructions. The 2^−ΔΔCt^ method was used to calculate relative gene expression with normalization to GAPDH [21]. The sequences of the primers used for qPCR are shown in Table S2.

### Transwell assays

The Transwell assay was used to assess the migratory and invasive capabilities of the cells according to previous studies [22]. The treated cells (8 × 10^4^) were seeded in the upper chambers of Transwell inserts (Millipore, Sandiego, CA, USA) in 200 μL of serum-free medium for migration or invasion assays. Subsequently, 800 μL of medium containing 15% FBS was introduced into the bottom chamber. Following incubation for 24 to 48 h, cells in the top chamber of the insert (nonmigrated cells) were removed with a cotton swab. Meanwhile, cells in the bottom chamber (migrated cells) were fixed in methanol, stained with 0.1% crystal violet, and then counted and imaged under an inverted microscope. In the cell invasion experiment, the top chamber of the insert was pre-coated with Matrigel (BD Biosciences, San Jose, CA, USA).

### Tube formation assays

Briefly, 60 μL of Matrigel was added to 96-well plates and then incubated in the cell incubator at 37 °C for 30 min. Subsequently, 2 × 10^4^ HUVECs in 100 μL of complete medium were added to the Matrigel-coated wells. After 24 h of transfection, 100 μL of treated cells supernatant was added to 96-well plates and, after 12 h, the HUVECs were exposed to the supernatant and allowed to grow. The resulting tubular structures were visualized and imaged under a microscope [23]. The numbers of meshes and branches were calculated using ImageJ.

### Nuclear and cytoplasmic fractionation

Following the manufacturer’s instructions, nuclear and cytoplasmic RNA was extracted using the Nuclear and Cytoplasmic Extraction Kit (Thermo Fisher Scientific, Waltham, MA, USA) following the manufacturer’s recommendations. The internal references for cytoplasmic and nuclear RNA were GAPDH, β-actin, and U6 RNA. qRT-PCR was used to quantify the RNA levels in both the cytoplasmic and nuclear compartments.

### Fluorescence in situ hybridization (FISH)

SW480 and LOVO cells were inoculated in 24-well plates, and when the cells grew to logarithmic growth phase, the medium was discarded and the cells were washed with PBS. Subsequently, the cells were fixed with 4% PFA paraformaldehyde, washed and added with prehybridization solution at 65 °C for 1 h. Then the hybridization solution containing the probe was added and reacted in the dark at 65 °C in a hybridizer for 48 h. The cells were washed with 4× and 2× SSC, and then subjected to DAPI staining. The slices were observed by fluorescence microscopy after sealed [23, 24]. RNA FISH probes were designed and synthesized by Ribobio (Guangzhou, China).

### Western blotting

Total protein was extracted from cells using RIPA lysis buffer (Beyotime, China). The proteins were then separated by SDS–PAGE and transferred to PVDF membranes. After blocking with 5% BSA (Beyotime), the membranes were incubated overnight at 4 °C with the relevant primary antibodies (anti-HMGCS2, CST, #20940; anti-IGF2BP1, Abcam, ab184305; anti-METTL16, Abcam, ab252420; and GAPDH, Beyotime, AF5009). After washing, the membranes were incubated with HRP-conjugated secondary antibodies. Protein bands were visualized using an ECL substrate and a fully automated chemiluminescence imaging analysis system. GAPDH was used as the internal reference [24].

### RNA pull-down and RNA immunoprecipitation (RIP)

Both full-length and shorter probes specific to LOC101928222, as well as negative control probes, were generated by Biotech. RNA pull-down assays were conducted with the Pierce Magnetic RNA-Protein Pull-down Kit (Thermo Fisher Scientific) according to the manufacturer’s instructions. Briefly, RNA was labeled with biotin using 5′- and 3′-RACE kits (Thermo Fisher Scientific) and co-incubated with magnetic beads at room temperature for approximately 30 min. Protein lysates were then obtained from the indicated cells using IP lysis buffer containing protease inhibitors. Previously washed magnetic beads were added to the protein lysates in a protein-RNA binding solution (Thermo Fisher Scientific) and incubated at 4 ℃ for 2 h. Subsequently, the proteins that co-precipitated with the magnetic beads were washed, collected, and subjected to western blotting, silver staining, and mass spectrometric analysis.

For the RIP assay, the Magna RIP Kit (Millipore, Sandiego, CA, USA) was used according to the manufacturer’s instructions. Approximately 3 × 10^7^ cells were lysed on ice in RIP lysis buffer and the lysates were immunoprecipitated with anti-IGF2BP1 (Abcam, ab184305) or IgG antibody (Millipore, Sandiego, CA, USA) and magnetic beads overnight at 4 °C. Samples of the immunoprecipitated RNA were purified and subjected to qRT-PCR [23].

### RNA stability assays

Transfected cells were treated with actinomycin D at a concentration of 10 μg/mL to block mRNA transcription and collected at several time points (0, 2, 4, 6, and 8 h). Total RNA extraction was performed using Trizol reagent, followed by qRT-PCR [25].

### Immunol fluorence staining (IF)

For the IF staining assay, Immunol Fluorence Staining Kit was used according to the manufacturer's instructions [26]. Clean coverslips were placed in six-well plates, seeded with cell culture overnight to make about 50%-80% full, cells were fixed with fixative for 30 min, followed by blocking with blocking solution for about 60 min, followed by the action of a specific primary antibody for 60 min, removal of the primary antibody, washed with washing solution and then added with 1mL of diluted fluorescently labeled secondary antibody for 60 min of light-avoidance incubation, washed with washing solution and then added with diluted fluorescence quenching sealing solution on slides. After the washing solution was washed, diluted anti-fluorescence quenching sealing solution was added to the slide, covered with a coverslip with cells attached, and finally photographed using confocal microscopy.

### MeRIP qRT-PCR

The Manga MeRIP m6A Kit was used for gene-specific m6A qPCR assays (Millipore) [25, 26]. Briefly, total RNA was extracted with Trizol. A total of 100 μg of RNA was sheared into fragments of <200 nt long using metal ion-induced fragmentation. The sheared RNA was purified and incubated with magnetic bead-conjugated m6A or IgG antibodies in IP buffer at 4 °C overnight. Methylated RNA eluted from the magnetic bead-conjugated immunoprecipitation complex was collected and further quantified by qRT-PCR.

### Cholesterol assays

CRC cells (1 ×10^6^) or animal tissues (2 mg) were harvested. Total cholesterol concentrations were evaluated by the Tissue Cell Total Cholesterol Enzymatic Assay Kit (Beijing, China) according to the manufacturer's instructions. Briefly, cells or tissues were collected by digestion, 0.1 mL of lysate was added per 1 × 10^6^ cells, and centrifugation was used to obtain supernatants. Subsequently, 10 µL of supernatant was taken from each well and added into a preprepared working solution, followed by incubation at room temperature for 20 min. The absorbance of each solution at 550 nm was measured using a microplate reader.

The treated CRC cells in logarithmic growth phase were inoculated in 6-well plates at a density of 5×10^5^ cells/well and treated with cholesterol at concentrations of 0, 5, 10 or 15 µM for 24 h for subsequent experiments.

### Tumor xenograft model

Animal experiments were approved by the Animal Care Ethics Committee of Nanjing Medical University. After 24 h of transfection, LOVO cells were collected and washed with HBSS. Subsequently, 1 μL of CM-DiI (Invitrogen, USA) was added to the cells, followed by incubation first for 5 min at 37 °C and then for 15 min at 4 °C. AB wild-type zebrafish embryos at 48 h post-fertilization (hpf) were fixed on a low-melting-point agarose gel and the previously labeled cells were introduced into the perivitelline space. On day 2 after tumor cell transplantation, the zebrafish were cultivated at 34 °C and then examined under a microscope. Successfully transplanted zebrafish embryos of comparable size were maintained at 34 °C until the conclusion of the experiment. At 4 days post-injection, the juveniles were fixed in low-melt gel for imaging [27].

Five-week-old male nude mice (BALB/c) mice divided into five per group were used for the xenograft tumor model and metastasis models. For the xenograft model, 100μL cell suspensions containing 1× 10^6^ CRC cells sin 100 µLPBS were injected into the armpits of the mouse limbs which divided into six groups (shNC, shHMGCS2, shLOC+OE-HMGCS2, shLOC, shHMGCS2+OE-IGF2BP1, shLOC+OE-GF2BP1). The tumor volumes were measured every 3 days. Tumor volumes were determined by caliper measurement (V = length × width^2^ × 0.5). Twenty-one days after the injection, all mice were sacrificed and the weights of the tumors were measured. For metastasis models, 100μL cell suspensions containing 1× 10^6^ CRC cells were injected into the tail veins or distal tip of the spleen or of mice. About five weeks later, D-luciferin (150 mg/kg) was intra-peritoneal injected into the mice and the metastases were visualized using an IVIS 100 Imaging System (Xenogen, USA). Then, the mice were excised to count the metastatic nodules in the liver or lung. The acquired tumor tissues were subjected to cholesterol assays or immunohistochemistry staining.

## Immunohistochemistry (IHC)

Procedures were similar to the previous study [26, 27]. In brief, the tissue slides were sectioned and then dewaxed with xylene, hydrated with alcohol, repaired with EDTA microwave heat for 5~8min, and cooled to room temperature. Subsequently, 5% BSA was added dropwise, incubated for 30min at room temperature, excess liquid was shaken off, primary antibody was added at 4℃ overnight, then HRP-labeled secondary antibody was added dropwise, and left at room temperature for 20min, DAB chromogen was added dropwise, and hematoxylin was used for re-staining after washing, and finally the slides were sealed and photographed.

### Statistical analysis

Statistical analysis was conducted using GraphPad Prism V8.0. The Student's *t*-test was used for comparisons between two groups, on the assumption that the data followed a normal distribution and the variances were equal. The corrected Student's *t*-test was used when the data obeyed normality but the variances were not equal. The nonparametric Wilcoxon rank sum test was applied when the data were not normally distributed. Comparisons among three or more groups were performed using one-way ANOVA. Studies were conducted in a manner that ensured independent replication, with each experiment being performed a minimum of three times. *P*-values <0.05 were deemed statistically significant.

## Results

### LOC101928222 is upregulated in CRC and associated with poor prognosis

**Fig. 1:**
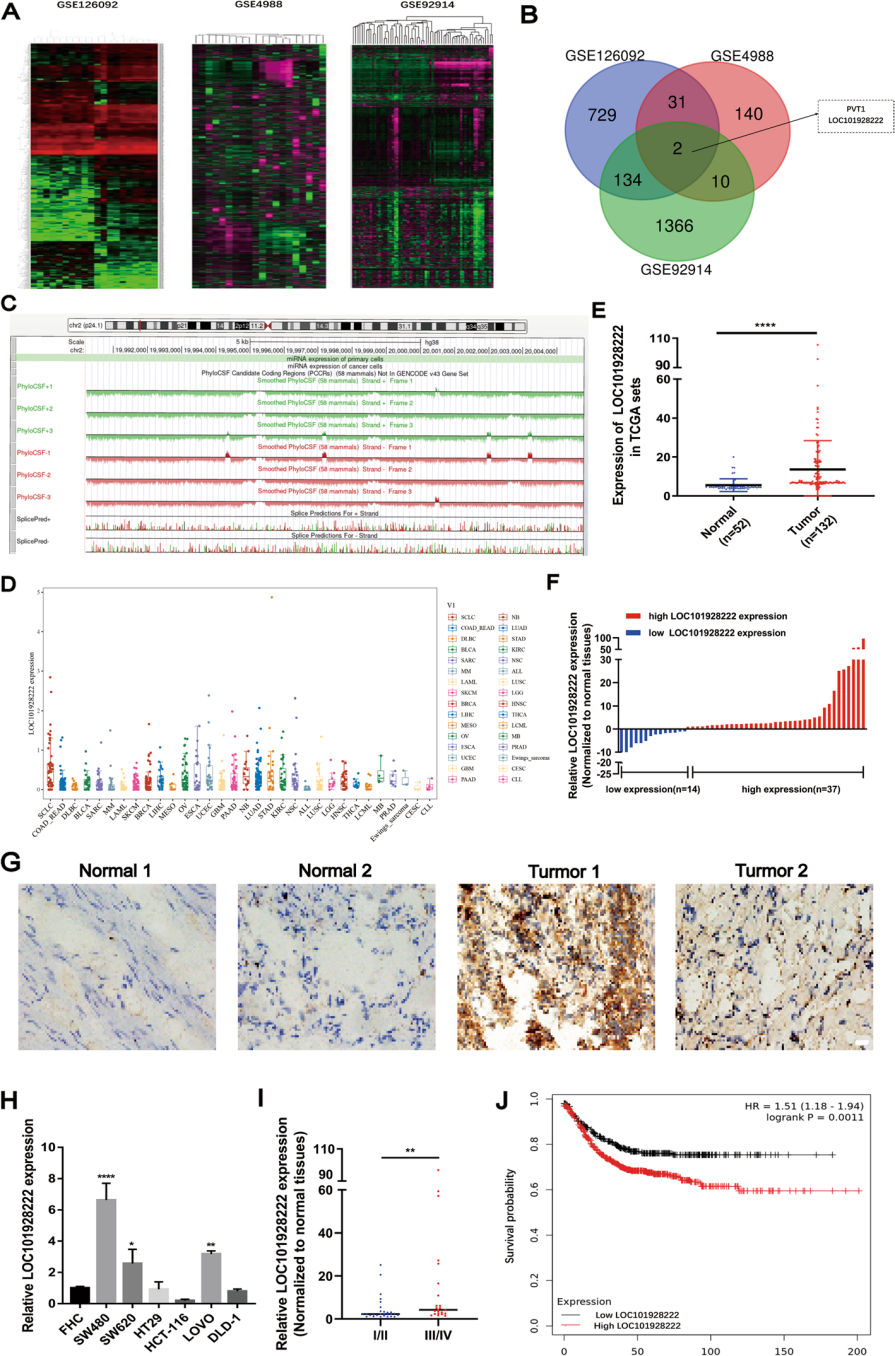
LOC101928222 is upregulated in CRC and associated with poor prognosis. **A** Differentially expressed genes were analyzed based on the GEO database (GSE126092, GSE4988, GSE92914). **B** Venn diagram showing overlap between differentially expressed lncRNAs. **C** The UCSC database shows the protein coding capacity of LOC101928222. **D** The overall expression of LOC101928222 in multiple human cancers at TCGA. **E** TCGA database showing expression levels of LOC101928222 in CRC tissues (*n*=132) and normal colorectal tissues (*n*=52). **F** The expression of LOC101928222 in 51 pairs of CRC tissues was detected and classified into relatively high-expression and low-expression group.** G** ISH analyses of LOC101928222 expression in colorectal cancer and adjacent normal tissues. Scale bar: 100 μm. **H** Expression levels of LOC101928222 in CRC cell lines and normal human colon epithelial cell was detected by qRT-PCR. **I** Relative Expression of LOC101928222 in Patients with higher Pathologic Staging (III/IV) and lower Pathologic Staging (I/II). **J** Kaplan-Meier analysis of survival curves in CRC patients with low LOC101928222 expression and high LOC101928222 expression. *p < 0.05, **p < 0.01, ***p < 0.001, ****p < 0.0001

To explore the potential lncRNAs affecting CRC progression, we performed a differential expression analysis by downloading the microarray data of “CRC tissues/corresponding normal-appearing tissue” (GSE126092), “blood sample of CRC patients/blood sample of healthy donor” (GSE4988), and “CRC primary tissues/liver metastasis tissues” (GSE92914) from the GEO database (Fig. 1A). We identified several differentially expressed lncRNAs, among which PVT1 and LOC101928222 were significantly upregulated in all three datasets (Fig. 1B). Because PVT1 has been reported more frequently in CRC, we selected LOC101928222 as our study subject. Then, the UCSC database showed that LOC101928222 lacked protein-coding capability (Fig. 1C). Analysis of TCGA public dataset showed that LOC101928222 was upregulated in CRC (Fig. 1D, E). Subsequently, we further analyzed the expression of LOC101928222 in 51 pairs of samples collected from CRC patients and found that, in 37 of these pairs, LOC101928222 expression was higher in CRC tissue than in normal surrounding tissue (Fig. 1F). In situ hybridization (ISH) of CRC tissue microarrays further validated the elevated LOC101928222 expression in CRC samples (Fig. 1G). Similarly, LOC101928222 expression was increased in SW480, SW620, and LOVO cells compared with that in FHC cells (Fig. 1H). Our analysis further indicated that there was significantly associated with the elevated expression of LOC101928222 and CRC pathological stage (III/IV), as well as poorer survival (Fig. 1I, J). In summary, these results indicated that LOC101928222 is upregulated in CRC and is associated with worse clinical outcomes.

### LOC101928222 promotes CRC progression in vitro and in vivo

**Fig. 2:**
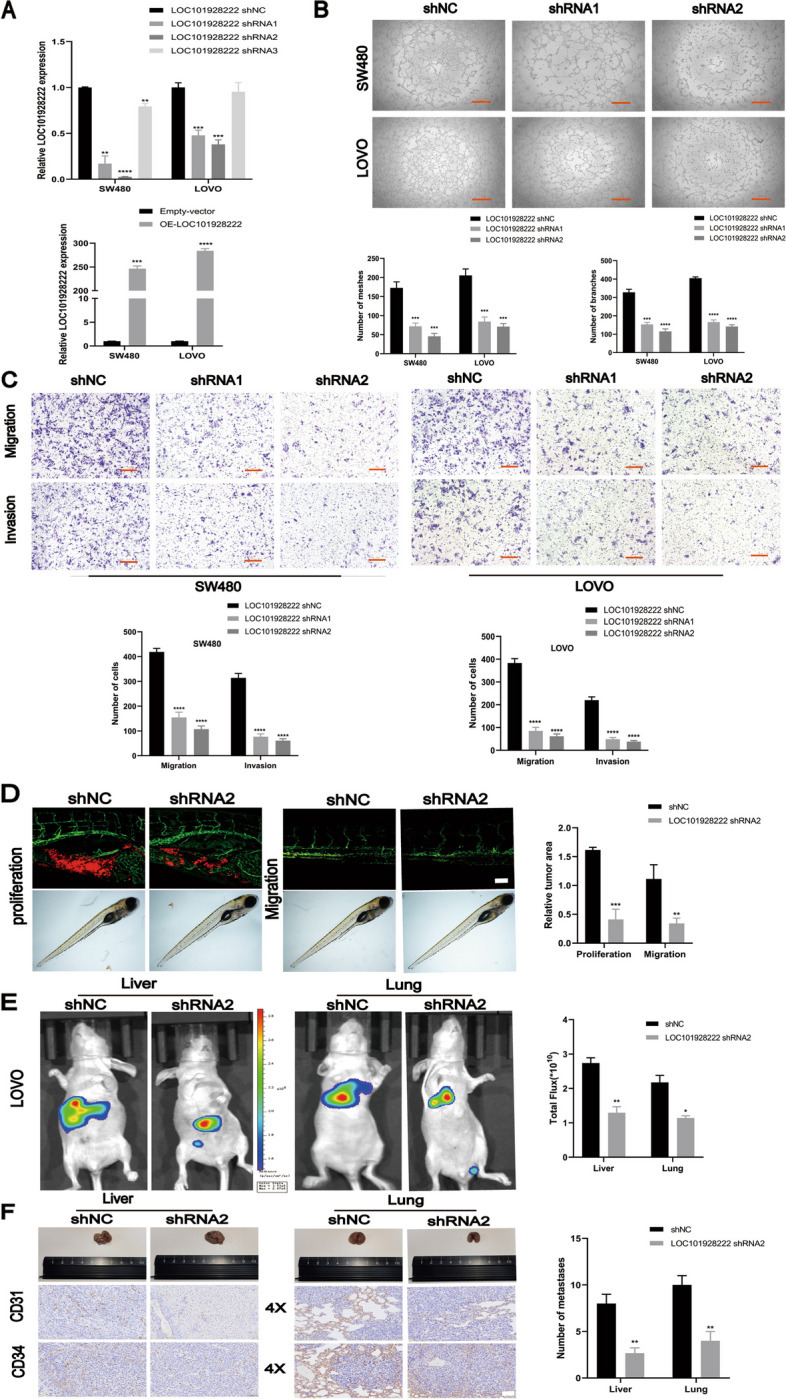
LOC101928222 promotes progression of CRC in vitro and vivo. **A** The knockdown and overexpression efficiency of LOC101928222 in SW480, LOVO cells were detected by qRT-PCR. **B** Tube formation assays were performed to detect the angiogenesis of HUVECs cells (number of branches, meshes), which co-cultured with supernatant of CRC cells transfected with LOC101928222 shRNAs. Scale bar: 100 μm. **C** Transwell assays were performed to determine the effects of LOC101928222 on migration and invasion in SW480 and LOVO cells transfected with LOC101928222 shRNAs. Scale bar: 50 μm. **D** Confocal imaging showing cells in CM-DiI-positive areas in the yolk (proliferation) and trunk(migration). **E** Images and analysis of luminescence intensity in metastasis models. **F** Images and IHC staining of metastatic tumors in the livers and lungs of mice. The number of metastases in livers or lungs was analyzed. Scale bar: 50 μm.*p < 0.05, **p < 0.01, ***p < 0.001, ****p < 0.0001

We and others have previously shown that tumor angiogenesis and tumor migration and invasion ability play crucial roles in CRC development, especially tumor metastasis [28, 29]. To further investigate the biological function of LOC101928222 in CRC, we designed a LOC101928222 overexpression vector and shRNAs. Next, shRNA1 and shRNA2 exhibited superior knockdown efficiency, thereby warranting their selection for subsequent experimental investigations (Fig. 2A). The effect of LOC101928222 on angiogenesis was then examined by the tube formation assay, which showed that the knockdown of LOC101928222 inhibited the tube-forming capability of HUVECs (Fig. 2B). Transwell assay results demonstrated that the knockdown of LOC101928222 reduced the migratory and invasive abilities of CRC cells (Fig. 2C). Then, to evaluate the ability of LOC101928222 to adjust CRC cell proliferation, CCK-8 and colony formation were performed. LOC101928222 knockdown inhibited the proliferation of SW480 and LOVO cells (Figure S1). Next, to explore the effect of LOC101928222 in vivo, xenograft tumor models and metastasis models were established. We found that LOC101928222 knockdown inhibited tumor growth and migration in vivo (Fig. 2D). Further, the liver and lungs of metastasis models were injected with stably transfected CRC cells via the distal tip of the spleen and tail veins of the nude mice, respectively. Thereafter, higher fluorescence intensity and larger numbers of metastasis nodules in the liver and lungs indicated that LOC101928222 facilitated metastasis. IHC showed that, compared with the control group, the shLOC101928222 group showed downregulated CD31 and CD34 expression (Fig. 2E, F). Thus, these results suggested that CRC cell tumorigenesis and metastasis could be promoted by upregulated LOC101928222 expression.

### LOC101928222 promotes CRC progression by regulating HMGCS2

**Fig. 3:**
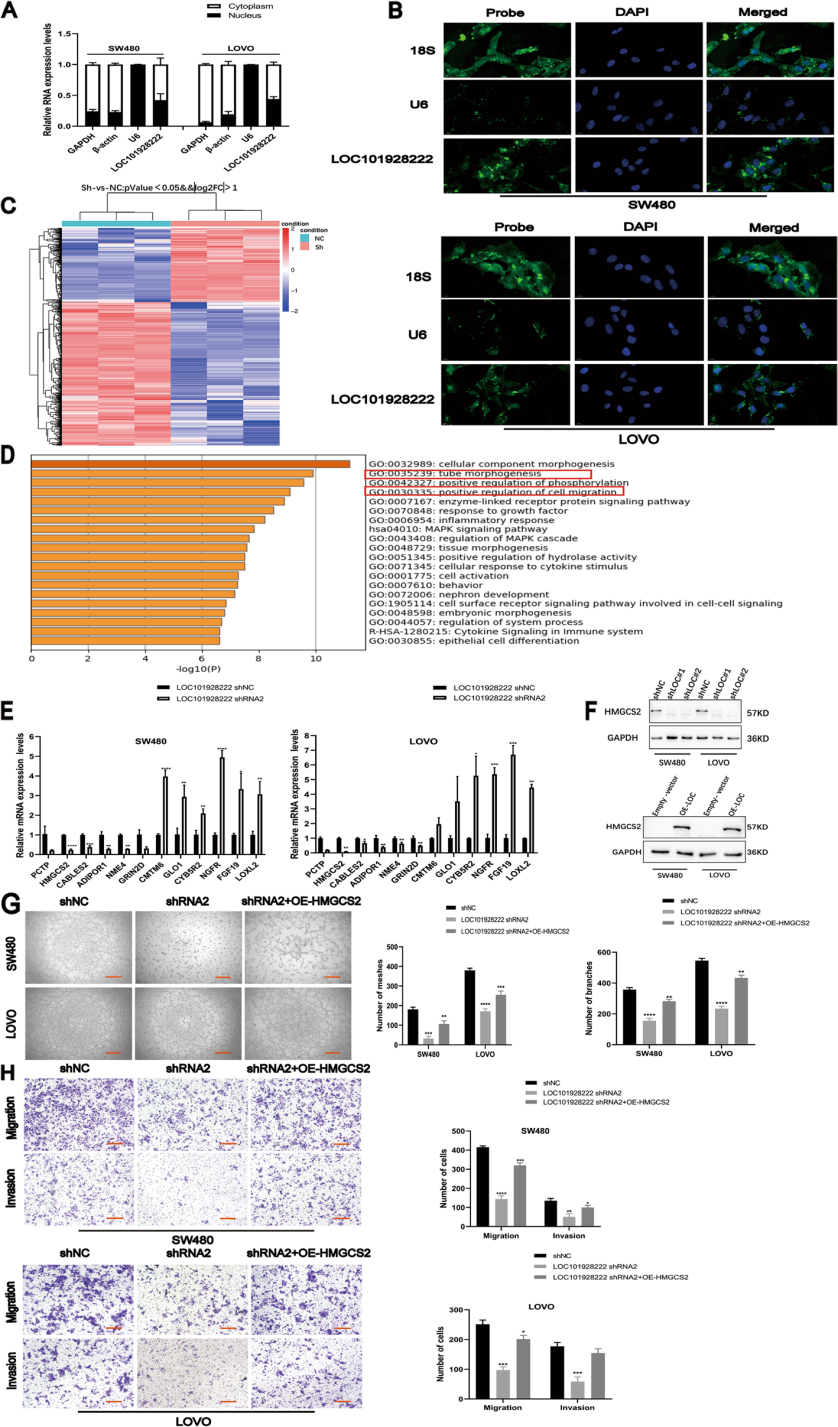
LOC101928222 promotes CRC progression by regulating HMGCS2 expression. **A** Subcellular localization of LOC101928222 in SW480 and LOVO cells. The nuclear control was U6, and the cytoplasm control was GAPDH, β-actin. **B** FISH analysis of the location of LOC101928222 in the cytoplasm and nuclear fractions of SW480 and LOVO cells. **C** Heatmap of differentially expressed mRNA identified by RNA-seq after LOC101928222 knockdown in LOVO cells. **D** Metascape website for enrichment analysis of differential genes. **E** qRT-PCR were performed to validate genes with high differential expression folds in the RNA-seq results. **F** Western blotting was used to detect changes in HMGCS2 protein levels after knockdown and overexpression of LOC101928222. **G** Tube formation rescue assays for effects of HMGCS2 overexpression on angiogenesis of SW480 and LOVO cells with LOC101928222 shRNA. Scale bar: 100 μm. **H** Transwell rescue assays for effects of HMGCS2 overexpression on migration and invasion of SW480 and LOVO cells with LOC101928222 shRNA. Scale bar: 50 μm.*p < 0.05, **p < 0.01, ***p < 0.001, ****p < 0.0001

It has been reported that lncRNAs contribute to the progression of tumorigenesis by regulating their neighboring genes [30]. As the localization of lncRNAs is closely related to the mechanism of their function, we first performed nuclear and cytoplasmic fractionation and FISH, which found that LOC101928222 was localized to both the nucleus and cytoplasm in SW480 and LOVO cells (Fig. 3A, B). To find potential LOC101928222 target genes, we performed RNA high-throughput sequencing and found that 458 genes that were upregulated and 236 were downregulated in the shNC group relative to the shLOC101928222 group (Fig. 3C). Gene enrichment analysis showed that LOC101928222 was significantly associated with tube formation and cell migration (Fig. 3D). We then selected the genes with the most significant differential expression in the RNA-seq results for further validation by qRT-PCR and found a high degree of conformity with the sequencing results (Fig. 3E). The analysis revealed the presence of HMGCS2 in both tube formation and cell migration pathways, and further experiments revealed that HMGCS2 was downregulated after LOC101928222 knockdown, but was upregulated following LOC101928222 overexpression (Fig. 3F). This demonstrated that HMGCS2 is a potential target gene of LOC101928222. We then performed rescue experiments and found that HMGCS2 overexpression reversed the LOC101928222 knockdown-mediated reduction in angiogenesis, cell migration, and cell invasion (Fig. 3G, H). These findings indicated that LOC101928222 can facilitate angiogenesis and metastasis in CRC via its regulatory effect on HMGCS2 expression.

### LOC101928222 regulates HMGCS2 expression by interacting with IGF2BP1

**Fig. 4:**
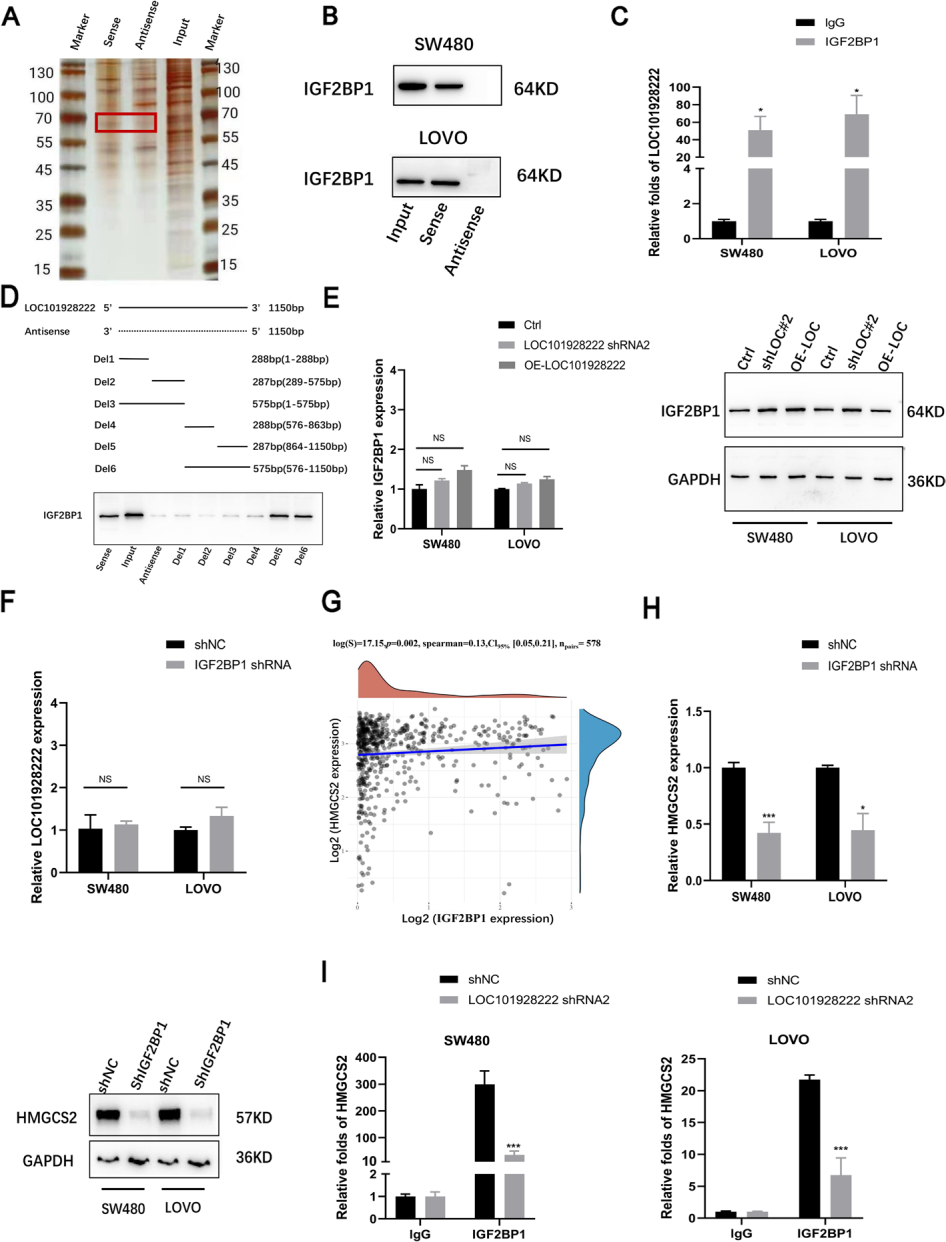
LOC101928222 interacts with IGF2BP1 to regulate HMGCS2 expression. **A** RNA pull-down was performed in LOVO cells lysates using antisense and sense LOC101928222, followed by silver staining. Bands showing clear changes in IGF2BP1 are shown. **B** Western blotting was used to detect the specific interaction between IGF2BP1 and LOC101928222. **C** RIP was performed to examine the enrichment between LOC101928222 and IGF2BP1 in SW480 and LOVO cells.** D** Western blotting was used to examine IGF2BP1 in the pull-down precipitates with segmented LOC101928222. **E** qRT-PCR and western blotting assays were performed to detect the effects of knockdown or overexpression of LOC101928222 on IGF2BP1 expression. **F** qRT-PCR were performed to detect the effect of knockdown of IGF2BP1 on LOC101928222. **G** Correlation between IGF2BP1 and HMGCS2 based on TCGA database. **H** qRT-PCR and western blotting assays were performed to detect the effects of knockdown of IGF2BP1 on HMGCS2 expression. **I** RIP assays were performed to detect the effect of knockdown of LOC101928222 on the enrichment of IGF2BP1 with HMGCS2. *p < 0.05, **p < 0.01, ***p < 0.001, ****p < 0.0001

We next explored how LOC101928222 affects HMGCS2 expression. Studies have shown that lncRNAs exert regulatory control on downstream genes by interacting with RNA-binding proteins (RBPs) [31]. Consequently, we performed RNA pull-down assay using LOVO cells to identify RBPs that interact with LOC101928222. According to silver staining, sense-LOC101928222 and antisense-LOC101928222 pulled down significantly different proteins of 55–70 kDa (Fig. 4A). The protein was identified as IGF2BP1 (64 kDa) by mass spectrometry. The interaction between LOC101928222 and IGF2BP1 in SW480 and LOVO cells was confirmed by RNA pull-down and western blot assays (Fig. 4B). Subsequent RIP assays also demonstrated the direct interaction between LOC101928222 and IGF2BP1 (Fig. 4C). To further determine which fragment of LOC101928222 binds to IGF2BP1, we performed deletion map analysis and RNA pull-down assays. We found that the LOC101928222 fragment Del5 (864–1150 bp) could bind to IGF2BP1 (Fig. 4D). Interestingly, we further observed that the knockdown or overexpression of LOC101928222 did not affect the expression of IGF2BP1 (Fig. 4E), similarly, IGF2BP1 did not affect LOC101928222 expression (Fig. 4F). This suggested that LOC101928222 may form a complex with IGF2BP1 to affect downstream target gene expression. Subsequent analysis revealed a positive correlation between IGF2BP1 and HMGCS2 (Fig. 4G). We then confirmed that the knockdown of IGF2BP1 affects HMGCS2 expression (Fig. 4H) and that HMGCS2 mRNA can interact with IGF2BP1, as detected using a RIP assay (Fig. 4I). The enrichment of HMGCS2 was reduced when LOC101928222 was knocked down (Fig. 4I), suggesting that LOC101928222 regulates HMGCS2 expression by binding to IGF2BP1.

### The LOC101928222/IGF2BP1 complex promotes CRC progression by stabilizing HMGCS2 mRNA

**Fig. 5:**
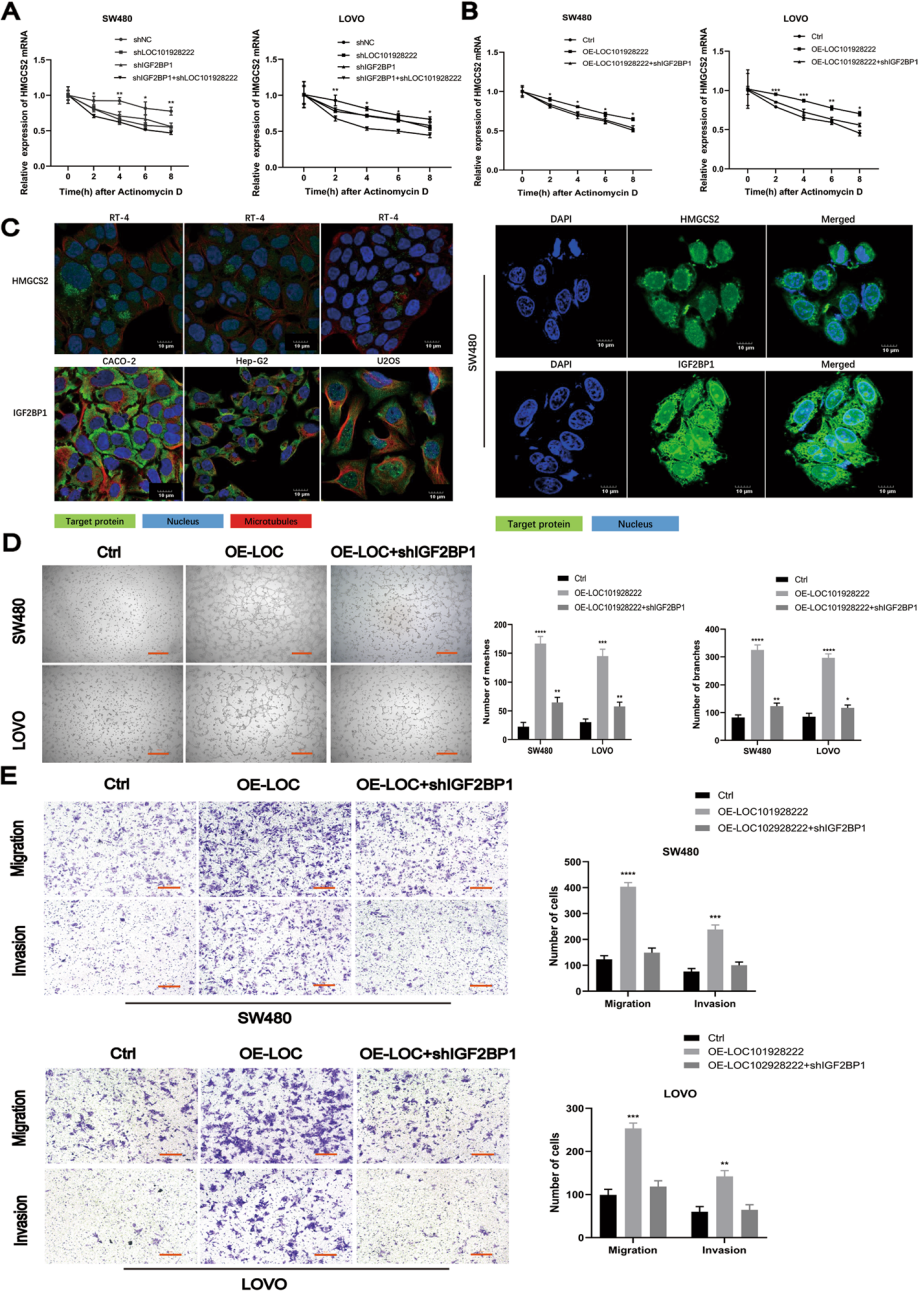
LOC101928222/IGF2BP1 complex promotes CRC progression by stabilizing HMGCS2 mRNA. **A** Changes in the stability of HMGCS2 mRNA after knockdown of LOC10192822, IGF2BP1, or both LOC10192822 and IGF2BP1 were examined in SW480 and LOVO cells.** B** HMGCS2 mRNA stability was assayed in negative control, LOC101928222-overexpressing with or without IGF2BP2-kockdown in SW480 and LOVO cells. **C** Different tumor cellular localization of HMGCS2 and IGF2BP1 based on The Human Protein Atlas. IF were performed to determine CRC cellular localization of HMGCS2 and IGF2BP1. **D** Tube formation rescue assays for effects of IGF2BP1 shRNA on angiogenesis of SW480 and LOVO cells with LOC101928222 overexpression. Scale bar: 100 μm. **E** Transwell rescue assays for effects of IGF2BP1 shRNA on migration and invasion of SW480 and LOVO cells with LOC101928222 overexpression. Scale bar: 50 μm.*p < 0.05, **p < 0.01, ***p < 0.001, ****p < 0.0001

IGF2BP1 acts as an m6A “reader,” and RBP stabilizes target mRNAs [32]. We therefore hypothesized that LOC101928222 may stabilize HMGCS2 mRNA by interacting with IGF2BP1. Subsequently, we knocked down LOC101928222 or IGF2BP1 and found that HMGCS2 mRNA stability was reduced. HMGCS2 mRNA stability was reduced to a greater extent when both LOC101928222 and IGF2BP1 were knocked down compared with that observed when LOC101928222 or IGF2BP1 were knocked down alone (Fig. 5A). The knockdown of IGF2BP1 attenuated the LOC101928222 overexpression-mediated increase in HMGCS2 mRNA stability (Fig. 5B). Based on The Human Protein Atlas and IF, we found that both HMGCS2 and IGF2BP1 were predominantly localized in the cytoplasm in CRC cells (Fig. 5C). We then performed rescue experiments, which showed that the knockdown of IGF2BP1 reduced the increase in angiogenesis, migration, and invasion resulting from LOC101928222 overexpression (Fig. 5D, E). In conclusion, we demonstrated that LOC101928222 enhanced the stability of HMGCS2 mRNA by cooperating with IGF2BP1, which ultimately promotes CRC progression.

### LOC101928222 synergizes with IGF2BP1 to stabilize HMGCS2 mRNA via a METTL16-mediated m6A-dependent pathway

**Fig. 6:**
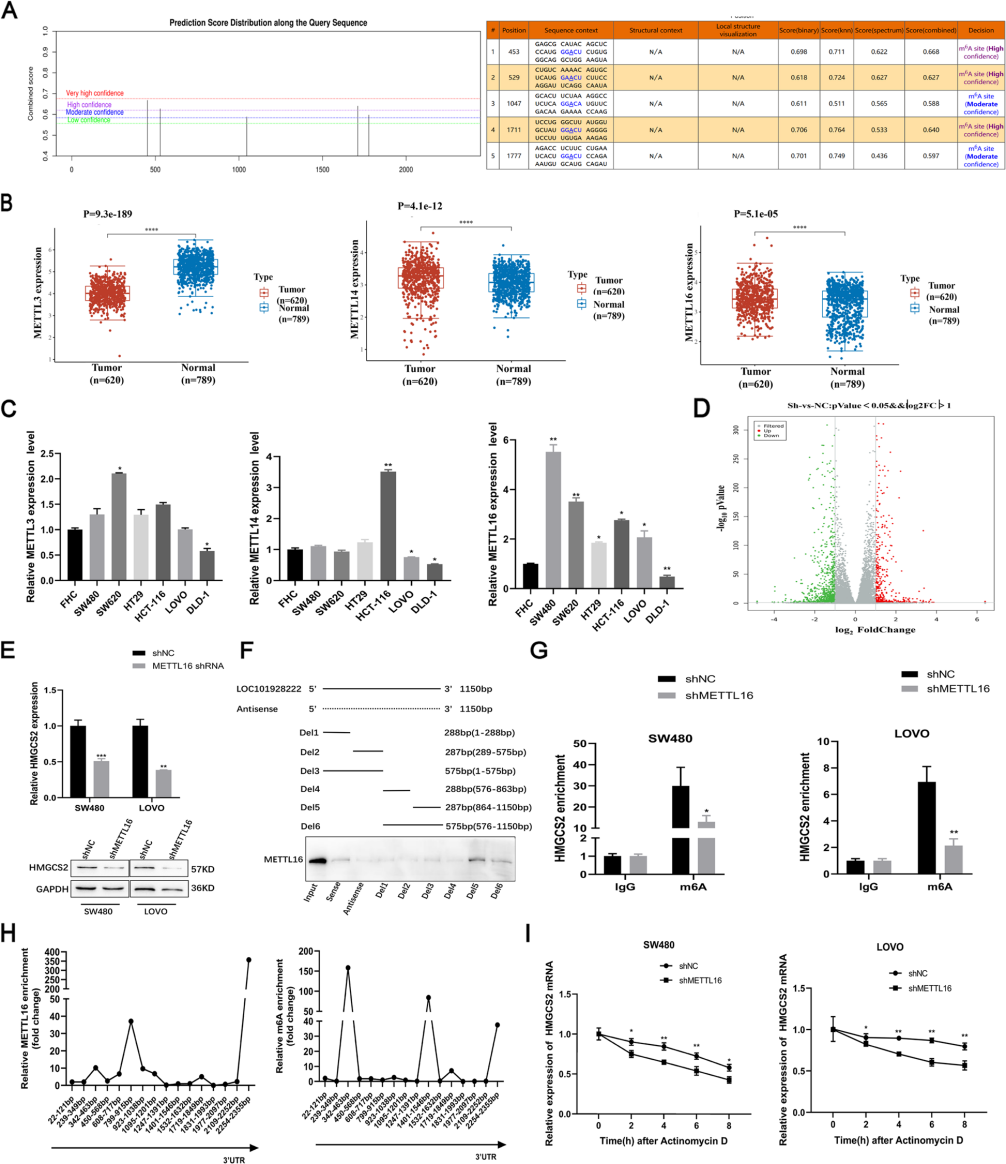
LOC101928222 synergizes with IGF2BP1 to stabilize HMGCS2 mRNA via a METTL16-mediated m6A-dependent pathway. **A** The SRAMP website predicts potential m6A modification sites in HMGCS2. **B** TCGA database showing expression levels of METTL3, METTL14 and METTL16 in CRC tissue (*n*=620) and normal colorectal tissue (*n*=789). **(C)** Expression levels of METTL3, METTL14 and METTL16 in CRC cell lines and normal human colon epithelial cell was detected by qRT-PCR. **D** Volcano plot of differentially expressed RNAs identified by RNA-seq after METTL16 knockdown in LOVO cells.** E** qRT-PCR and western blotting assays were performed to detect the effects of knockdown of METTL16 on HMGCS2 expression. **F** Western blotting was used to examine METTL16 in the pull-down precipitates with segmented LOC101928222. **G** MeRIP-qPCR was performed on SW480 and LOVO cells to detect changes in m6A levels in HMGCS2 after knockdown of METTL16. **H** MeRIP-qPCR was conducted to determine specific locations of HMGCS2 mRNA enrichment for METTL16 and m6A.** I** Changes in the stability of HMGCS2 mRNA after knockdown of METTL16 were examined in SW480 and LOVO cells. *p < 0.05, **p < 0.01, ***p < 0.001, ****p < 0.0001

N6-methyladenosine (m6A) is the predominant intramolecular alteration seen in RNA within eukaryotic cells. Over recent years, increasing evidence has indicated that m6A is involved in various aspects of RNA regulation within tumor cells. Specifically, m6A has been shown to influence post-transcriptional processes such as RNA splicing, translocation, stability, and protein translation. These effects are achieved through the addition, removal, or recognition of m6A-modified sites in RNA, facilitated by specialized enzymes known as “writers,” “erasers,” and “readers” [1, 33–35]. The “writers” complex, consisting mostly of METTL3, METTL14, and METTL16, is a crucial component of the dynamic process of methylation [33, 36]. To further explore whether m6A modification exists in HMGCS2, we utilized the SRAMP website assessment to identify five potential m6A modification sites in HMGCS2 (Fig. 6A). We then analyzed the expression of METTL3, METTL14, and METTL16 in CRC, and found that METTL14 and METTL16 are highly expressed in CRC (Fig. 6B). We then examined METTL3, METTL14, and METTL16 expression in CRC cell lines and found that the expression of METTL16 was higher than that of METTL3 and METTL14 (Fig. 6C). Interestingly, we knocked down METTL16 for high-throughput sequencing in LOVO cells and found that HMGCS2 was a potential target gene of METTL16 (Fig. 6D). Further validation revealed that the knockdown of METTL16 reduced HMGCS2 expression (Fig. 6E). We then performed deletion map analysis and RNA pull-down assays and found that the LOC101928222 fragment Del1 (864–1150 bp) binds to METTL16 (Fig. 6F). The findings obtained from the MeRIP studies demonstrated that the depletion of METTL16 reduced the enrichment of m6A methylation in HMGCS2 (Fig. 6G). MeRIP-qPCR experiments were then performed and confirmed that m6A modifications were predominantly present in the 3′-UTR of HMGCS2 mRNA (Fig. 6H). Finally, our assay revealed a significant decrease in HMGCS2 mRNA stability after the knockdown of METTL16 (Fig. 6I). Taken together, these data suggested that LOC101928222 synergizes with IGF2BP1 to stabilize HMGCS2 mRNA via METTL16-mediated m6A methylation in the 3′-UTR of HMGCS2.

### LOC101928222 mediates an increase in cholesterol levels in CRC, leading to its progression and the clinical relationships of related molecules

**Fig. 7:**
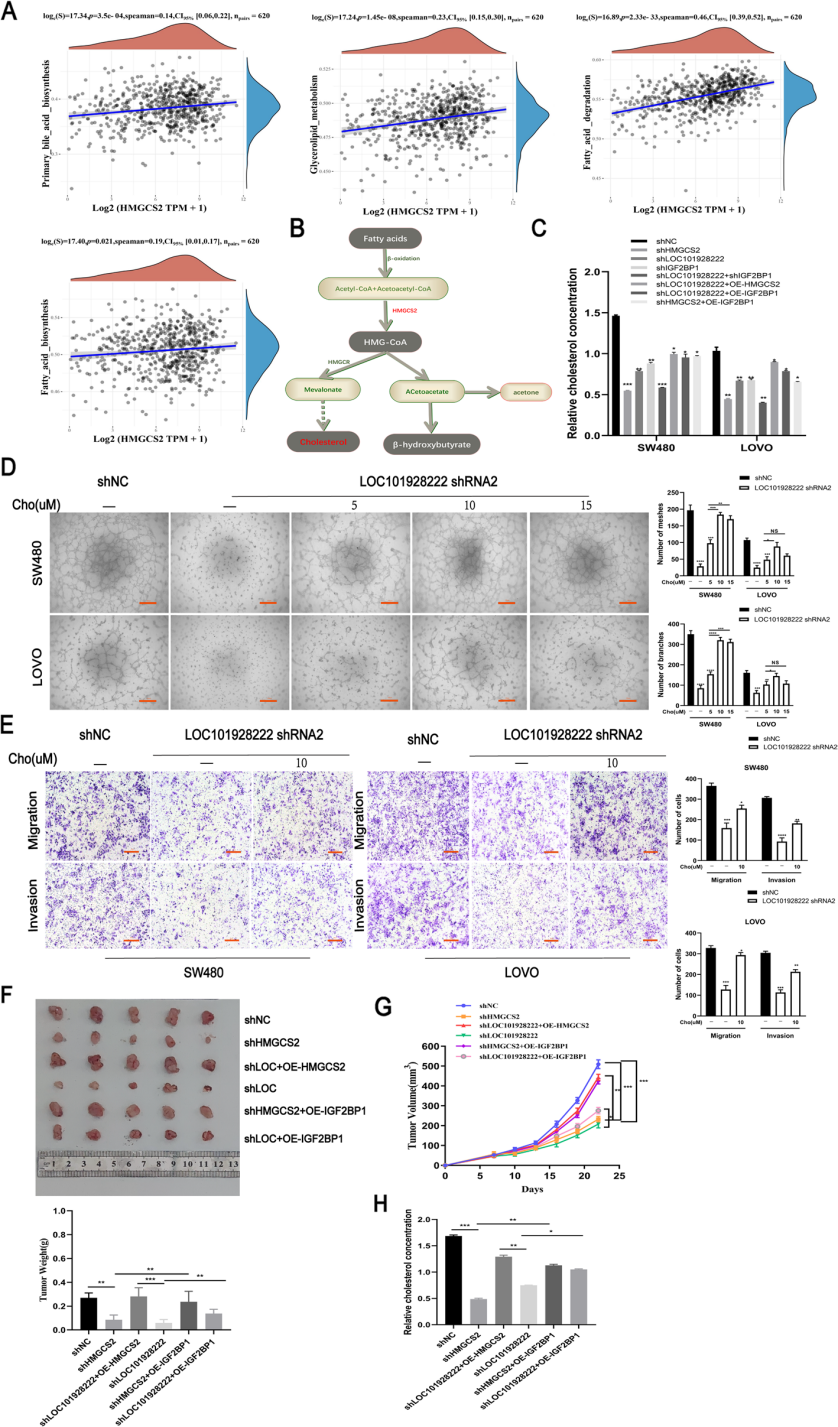
LOC101928222 mediates cholesterol increase in CRC leading to its progression. **A** Pathway correlation analysis of HMGCS2 based on TCGA database. **B** Simplified overview of cholesterol biosynthesis metabolism. **C** Cholesterol assays were performed to detect the effect of shNC,shHMGCS2,shLOC101928222,shlGF2BP1,shLOC101928222+shIGF2BP1,shLOC101928222+OE-HMGCS2,shLOC101928222+OE-IGF2BP1,shHMGCS2+OE-IGF2BP1 on cholesterol. **D** Tube formation rescue assay for the effect of 0, 5, 10 or 15 µM cholesterol (labeled Cho) on angiogenesis in SW480 and LOVO cells with knockdown of LOC101928222. Scale bar: 100 μm.** E** Transwell rescue assays for the effect of 0 or 10 µM cholesterol (labeled Cho) on migration and invasion in SW480 and LOVO cells with knockdown of LOC101928222. Scale bar: 50 μm. **F-G**. LOVO cells of the corresponding group (1× 10^6^/mouse) were injected into mice, and pictures of tumors **(F)**, tumor growth curve and tumor weight** (G)** of each mouse group were measured. **H** Cholesterol concentrations in mice tumors of each experimental group were measured. *p < 0.05, **p < 0.01, ***p < 0.001, ****p < 0.0001

There is increasing evidence supporting that abnormal cholesterol metabolism contributes to cancer progression by providing energy through macromolecular and cholesterol-mediated signaling [37]. Studies have suggested that HMGCS2 is closely associated with cholesterol metabolism in tumors [38, 39]. Accordingly, we performed a pathway correlation analysis of HMGCS2 in CRC and found that HMGCS2 was closely associated with lipid and cholesterol metabolism pathways (Fig. 7A, FigureS3). Furthermore, HMGCS2 is part of the cholesterol biosynthesis pathway (Fig. 7B). We subsequently found that LOC101928222 or HMGCS2 or IGF2BP1 knockdown reduced cholesterol synthesis in CRC cells, and overexpression of HMGCS2 or IGF2BP1 could rescue the decrease cholesterol synthesis of LOC101928222 or HMGCS2-knockdown (Fig. 7C). To demonstrate the impact of cholesterol on CRC angiogenesis, various doses of cholesterol were administered to CRC cells, with the results showing that cholesterol treatment reversed the reduction in angiogenesis induced by LOC101928222 knockdown in a dose-dependent manner, with 10 µM cholesterol exerting the greatest effect (Fig. 7D). We then performed Transwell rescue experiments and found that 10 µM cholesterol also reversed the reduction in migration and invasion induced by LOC101928222 knockdown (Fig. 7E). Finally, we tested the pro-tumorigenesis function of related gene in vivo. As shown in Fig. 7F, G, LOC101928222 or HMGCS2 knockdown reduced tumor-initiating capacity of CRC cells in nude mice, overexpression of HMGCS2 or IGF2BP1 could rescue the tumor-initiating capacity of LOC101928222 or HMGCS2-knockdown CRC cells. And the concentration of cholesterol the corresponding changes in line with the tumor growth in each group of xenografts (Fig. 7H). The above results suggested that LOC101928222 induces an increase in cholesterol contents in CRC through HMGCS2, leading to CRC progression.

**Fig. 8:**
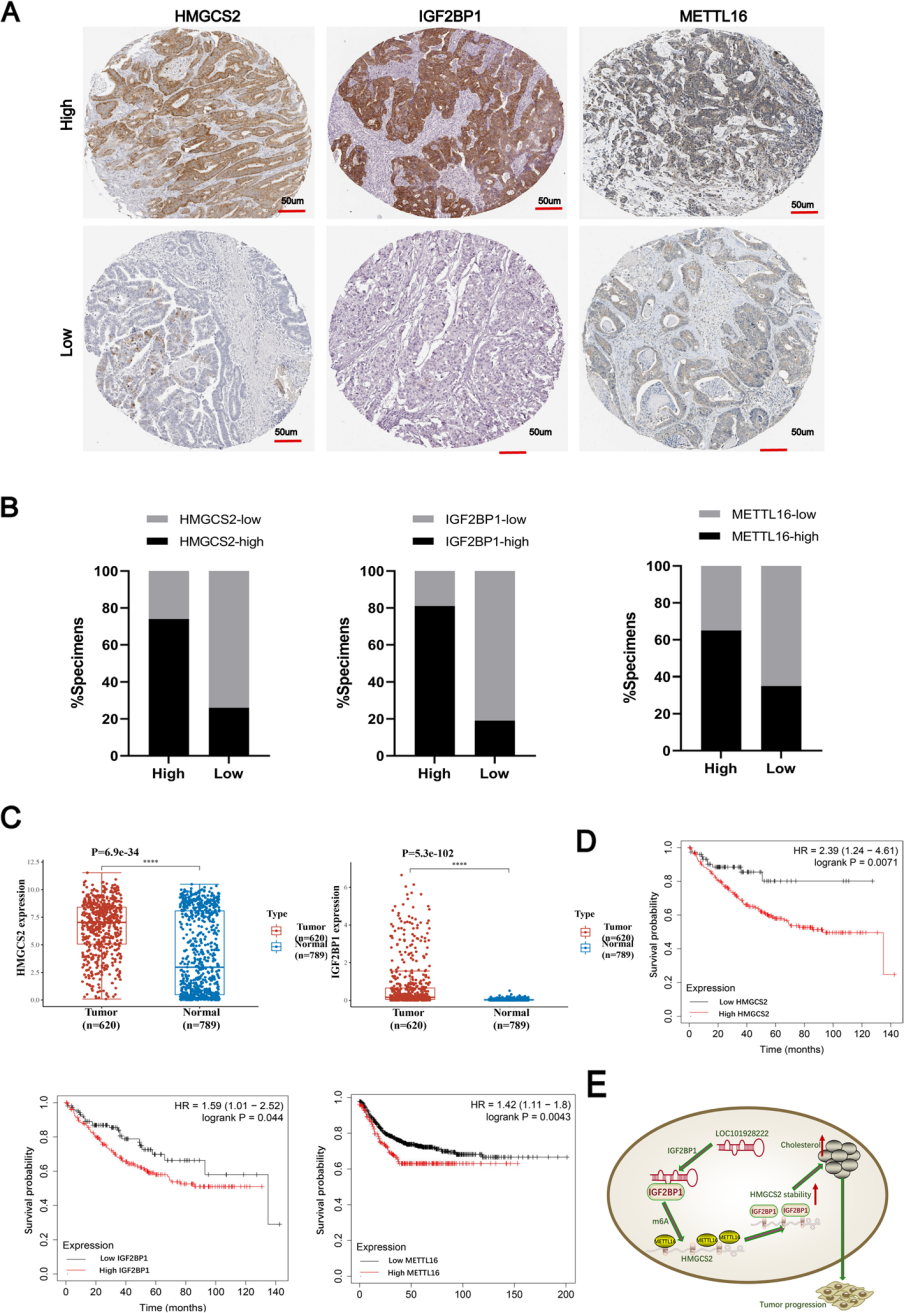
Clinical relationships of related molecules. **A** Representative images showing high or low expression of HMGCS2, IGF2BP1 and METTL16 in CRC patients’ tumor tissues. Scar bar: 50 μm.** B** Percentages of specimens showing different levels of HMGCS2, IGF2BP1 and METTL16 in 51 pairs of CRC tissues.** (C)**TCGA database showing expression levels of HMGCS2 and IGF2BP1 in CRC tissue (*n*=620) and normal colorectal tissue (*n*=789). **D** Kaplan-Meier analysis of survival curves in CRC patients based on HMGCS2, IGF2BP1 and METTL16 expression levels. **E** Schematic model depicting that LOC101928222 synergizes with IGF2BP1 to stabilize HMGCS2 mRNA through an m6A-dependent pathway, leading to increased cholesterol synthesis and ultimately promoting the development of CRC. * *p* < 0.05, **p < 0.01, ***p < 0.001, ****p < 0.0001

HMGCS2, IGF2BP1, and METTL16 were found to be highly expressed overall in CRC tissue, as determined by immunohistochemistry (Fig. 8A, B). Subsequent analysis suggested that HMGCS2 and IGF2BP1 were upregulated in CRC (Fig. 8C). Kaplan-Meier survival curve analysis revealed that high expression of HMGCS2, IGF2BP1, and METTL16 was significantly correlated with poor prognosis in patients with CRC (Fig. 8D). Overall, these results demonstrated that LOC101928222 synergizes with IGF2BP1 to stabilize HMGCS2 mRNA through an m6A-dependent pathway, leading to increased cholesterol synthesis, and, ultimately, the promotion of CRC development (Fig. 8E).

## Discussion

Over recent years, there has been a notable rise in the incidence and fatality rates associated with CRC [1]. Currently, the primary approach for treating metastatic CRC involves a combination of chemotherapy and targeted therapy [4]. However, owing to the complexity of CRC-associated metastatic mechanisms, the survival rate of CRC patients remains bleak. Progress relating to CRC etiology, diagnosis, and therapy is now experiencing a bottleneck. The recent emergence of epigenetic transcriptomics and tumor metabolism has presented novel avenues of investigation in the field of CRC diagnosis and therapy [7, 14, 40]. In the present study, we showed for the first time that LOC101928222, a recently identified lncRNA, exhibits significant upregulation in both CRC patients and CRC cell lines. Additionally, we elucidated the pivotal function of LOC101928222 in tumor growth and lipid metabolism. Further mechanistic studies suggested that LOC101928222 synergizes with IGF2BP1 to stabilize HMGCS2 mRNA via a METTL16-mediated m6A-dependent pathway, leading to increased cholesterol synthesis, and, consequently, the promotion of CRC development.

Tumor angiogenesis is a complex process, and abnormalities in angiogenesis are a key feature in tumor metastasis [41]. It is now recognized that the best way to effectively inhibit tumor growth and prevent metastasis is to cut off the lifeblood of the tumor, that is, to disrupt tumor angiogenesis [42]. An increasing body of evidence has indicated that lncRNAs have a significant impact on tumor angiogenesis [43–45]. A case in point is seen with JAG1, which stimulates angiogenesis in triple-negative breast cancer by facilitating the release of the exosomal lncRNA MALAT1 [43]. One study showed that H19 effectively recruited the m6A reader YTHDF1, which significantly promoted the translation of SCARB1 and facilitated angiogenesis, specifically in gastric cancer [44]. The lncRNA ZNRD1-AS1 was reported to promote the proliferation, migration, and angiogenesis of malignant lung cells via the miR-942/TNS1 signaling pathway [45]. However, the effect of lncRNAs on CRC angiogenesis has remained largely unknown. In this study, using bioinformatics analysis, we identified LOC10192822 as being highly expressed in CRC, and further found that high levels of LOC101928222 in CRC patients are associated with a poor prognosis. Importantly, we found that LOC101928222 exerts its pro-angiogenic effects in CRC by increasing cholesterol synthesis.

Metabolic reprogramming is a marker of malignancy. Cancer cells can reprogram their metabolism to support their progression by supplying adequate ATP generation and tumor building blocks [46]. It is increasingly clear that glycolysis, lipid metabolism, and amino catabolism are involved in tumor growth and metastasis [47, 48]. Recent studies on CRC metabolism have shown that cholesterol promotes the self-renewal of CRC stem cells [49], while FTO and ALKBH5 were reported to affect glycolysis by regulating HK2 expression in CRC [50]. Furthermore, increased cholesterol synthesis is crucial for the development of CRC, and correlates with patient prognosis [14–16]. However, its relevance in CRC angiogenesis has not been reported. Interestingly, HMGCS2 was identified as a LOC101928222-target gene by RNA-seq. HMGCS2 (3-hydroxy-3-methylglutaryl-CoA synthase 2) plays a key role in cholesterol synthesis as well as an oncogenic role in tumors [51, 52]. In prostate cancer, HMGCS2 facilitates tumor progression by promoting cholesterol biosynthesis [51]. In breast cancer, meanwhile, HMGCS2 promotes cell growth by augmenting mitochondrial oxidative stress and has emerged as a potential pharmacological target in this malignancy [52]. However, the precise function of HMGCS2 in CRC remains unclear. In this study, we revealed that LOC101928222 regulates cholesterol synthesis by stabilizing HMGCS2 mRNA, which contributes to angiogenesis in CRC. However, HMGCS2 may also affect ketone body production in fatty acid metabolism [38]. Whether HMGCS2 influences CRC metastasis through similar mechanisms requires further investigation.

The functions of lncRNAs are mainly reliant on their subcellular distribution. Studies have shown that lncRNAs are distributed in the nucleus and cytoplasm of tumor cells, and tend to interfere with the transcription and replication of target genes in the nucleus through chromatin modification and DNA methylation, and also play a role in the cytoplasm through regulating post-transcriptional modifications and protein translation [53–55]. In this study, we confirmed that LOC101928222 is mainly localized in the cytoplasm. Subsequent exploration revealed that LOC101928222 can interact with IGF2BP1 (insulin-like growth factor 2 mRNA-binding protein 1), a member of the insulin-like growth factor-2 mRNA-binding protein family (IGF2BP1, IGF2BP2, and IGF2BP3) that are involved in the regulation of RNA translocation, localization, translation, and stability [32, 56, 57]. The interaction between RPS15 and the K homology domain of IGF2BP1 is responsible for the recognition and direct binding of MKK3 and MAPK14 mRNA. This interaction promotes the translation of key proteins involved in the p38 MAPK pathway. In addition, RPS15 is involved in the development of metastasis and proliferation in human esophageal squamous cell carcinoma [56]. The IGF2BP1 protein selectively interacts with c-Myc mRNA, leading to enhanced mRNA stability and increased levels of c-Myc mRNA, hence facilitating the development of tumors [57]. We found that LOC101928222 and IGF2BP1 interact to regulate HMGCS2 mRNA stability. IGF2BP1 serves as a reader of m6A, and the 3′-UTR region of HMGCS2 exhibits a robust m6A modification signal. We found that the knockdown of METTL16 affects LOC101928222-induced HMGCS2 expression, suggesting that the LOC101928222/IGF2BP1 complex can stabilize HMGCS2 mRNA by recognizing METTL16-induced m6A modifications. However, the m6A modification status may also be influenced by the activity of other m6A methyltransferases or m6A demethylases. Further investigations are required to determine whether there are additional dysregulated methyltransferases or demethylases in CRC that are crucial for the m6A modification of HMGCS2.

## Conclusions

In summary, our study demonstrated the impact of LOC101928222 on angiogenesis, migration, and invasion in CRC. The effects of this gene were evaluated by a series of in vitro and in vivo experiments, as well as bioinformatics analysis. Furthermore, we demonstrated the clinical relevance of LOC101928222. In addition, from the epigenomic perspective, we found that LOC101928222 synergized with IGF2BP1 to stabilize HMGCS2 mRNA via a METTL16-mediated m6A-dependent pathway, leading to an increase in cholesterol synthesis, which consequently promoted CRC development. Our findings contributed to a more in-depth understanding of the molecular mechanisms underlying CRC metastasis and may facilitate the development of more effective and personalized therapeutic strategies for CRC.

### Supplementary Information

Additional file 1: Table S1. Sequences of all the shRNA.

Additional file 2: Table S2 Primer sequences for qRT-PCR

Additional file 3: Figure S1. LOC10192822 promotes colorectal cancer (CRC) cell proliferation in vitro. (A) For LOC101928222 knockdown, colony forming assays assessed CRC cell proliferation. (B) For LOC10192888 knockdown, the Cell Counting Kit (CCK)-8 assay assessed CRC cell viability. * *p*< 0.05, **p < 0.01, ***p < 0.001, ****p < 0.0001.

Additional file 4: Figure S2. The interference efficiency of METTL16 were verified by qRT-PCR in SW480 and LOVO cells.

Additional file 5: Figure S3. Bioinformatics analysis of the relationship between HMGCS2 and cholesterol metabolism.

## Data Availability

The data in the current study are available from the corresponding author on reasonable request.

## Abbreviations

CRC: Colorectal cancer
lncRNA: Long noncoding RNAs
GAPDH: Glyceraldehyde-3-Phosphate Dehydrogenase
HMGCS2: 3-hydroxy-3-methylglutaryl-CoA synthase 2
IGF2BP1: Insulin like growth factor 2 mRNA binding protein 1
METTL16: Methyltransferase 16, RNA N6-adenosine
METTL14: Methyltransferase 14, N6-adenosine-methyltransferase non-catalytic subunit
METTL3: Methyltransferase 3, N6-adenosine-methyltransferase complex catalytic subunit
m6A: N6-methyladenosine
ISH: In situ hybridization
qRT-PCR: Quantitative reverse transcription polymerase chain reaction
FISH: Fluorescence in situ hybridization
WB: Western blot
IHC: Immunohistochemistry
IF: Immunofluorescence
shRNA: Short hairpin RNA
RIP: RNA immunoprecipitation
UTR: Untranslated region
CHX: Cycloheximide
