# Supplementary material for: A novel lncRNA LOC101928222 promotes colorectal cancer angiogenesis by stabilizing HMGCS2 mRNA and increasing cholesterol synthesis
Source: J Exp Clin Cancer Res. 2024 Jul 4;43:185. doi: 10.1186/s13046-024-03095-8 (PMC11223299; doi:10.1186/s13046-024-03095-8)
Supplement: Supplementary file 1 — Additional file 1: Table S1. Sequences of all the shRNA. [file 13046_2024_3095_MOESM1_ESM.docx]

**Table S1 Sequences of all the shRNA**

| Name | Sequence ( 5' → 3' ) |
| --- | --- |
| sh-LOC101928222 -1 sense | GAUCCCAGCCAAGUCUGACUGCAAUTT |
| sh-LOC101928222 -1 antisense | AUUGCAGUCAGACUUGGCUGGGAUCTT |
| sh-LOC101928222 -2 sense | CGAAUGCCAAGUAGAUGCCACCAAATT |
| sh-LOC101928222 -2 antisense | UUUGGUGGCAUCUACUUGGCAUUCGTT |
| sh-LOC101928222 -3 sense | CAGAGAUCAAUAAAUGCAUGCUGAATT |
| sh-LOC101928222-3 antisense | UUCAGCAUGCAUUUAUUGAUCUCUGTT |
| sh-IGF2BP1 sense | ACGCUUAGAGAUUGAACAUUCTT |
| sh-IGF2BP1 antisense | GAAUGUUCAAUCUCUAAGCGUTT |
| sh-METTL16 sense | UCCCUUGAGACUCAACUAUAUTT |
| sh-METTL16 antisense | AUAUAGUUGAGUCUCAAGGGATT |
| sh-HMGCS2 sense | CCAGGAUUGGUGCCUUCUCUUTT |
| sh-HMGCS2 antisense | AAGAGAAGGCACCAAUCCUGGTT |
