# Supplementary material for: A novel lncRNA LOC101928222 promotes colorectal cancer angiogenesis by stabilizing HMGCS2 mRNA and increasing cholesterol synthesis
Source: J Exp Clin Cancer Res. 2024 Jul 4;43:185. doi: 10.1186/s13046-024-03095-8 (PMC11223299; doi:10.1186/s13046-024-03095-8)
Supplement: Supplementary file 2 — Additional file 2: Table S2 Primer sequences for qRT-PCR [file 13046_2024_3095_MOESM2_ESM.docx]

**Table S2 Primer sequences for qRT-PCR**

| Gene | Forward (5’-3’) | Reverse (5’-3’) |
| --- | --- | --- |
| LOC101928222 | ATGGAGTGGGAGGGTGATTT | AGGACACTGGGCTTTCACAA |
| GAPDH | GAAGGTGAAGGTCGGAGTC | GAAGATGGTGATGGGATTTC |
| β-actin | GAAGGTGAAGGTCGGAGTC | GAAGATGGTGATGGGATTTC |
| MALAT1 | GCGTTGTGCGTAGAGGAT | AAACCTACAACACCCGGAAA |
| PCTP | CCTTTTCCCATGTCCAACAG | CACCCGGGTTATCGAAGTAA |
| HMGCS2 | CCAAGGCTTCCCTTTACCTC | GCATCCTGGGATACTCGAAA |
| CABLES2 | CCTCATCTTTGCGTCGTACA | ATCTCCCGCTTTAAGCTCCT |
| ADIPOR1 | GAAGCTGACACGGTGGAACT | AGTGTCAGTACCCGCACCTC |
| NME4 | CAGGTCTGGGAAGGGTACAA | CTGGAACCACAGCTGGATCT |
| GRIN2D | CTCGGTCGCTTTTCCCTACT | TCGCAAATAAGGGTTGGATT |
| CMTM6 | AAGCTCCGCTTGTTCTGAGG | TTCTGGTCCCAAGTTCCCCT |
| GLO | GGTCCCGTCGTCTGTGATAC | TCACTCGTAGCATGGTCTGC |
| CYB5R2 | CCTGAATTCTTCCTTCCCCAGCA | CAACACGTCGTCGAACTGGG |
| NGFR | CCCGAGCACATAGACTCCTT | ACTGCACAGACTCTCCACGA |
| FGF19 | GCACAGTTTGCTGGAGATCA | ATCTCCTCCTCGAAAGCACA |
| LOXL2 | CTGTCCCCGCAGTAAAGAAG | ACGTGGCATTCGTTCAGACT |
| IGF2BP1 | AGCTTTACATCGGCAACCTC | GGCACCGAATGTTCAATCTC |
| METTL3 | GCTGACCATTCCAAGCTCTC | ATTTCTTGGCTGGCTCCTTT |
| METTL14 | GATAGCCGCTTGCAGGAGAT | TTTAACACGGCACCAATGC |
| METTL16 | TGAGAGGTGGCTGTTGGTC | AGTGAGCTAAGATCGCACCA |
