# Supplementary figures and images for: A novel lncRNA LOC101928222 promotes colorectal cancer angiogenesis by stabilizing HMGCS2 mRNA and increasing cholesterol synthesis
Source: J Exp Clin Cancer Res. 2024 Jul 4;43:185. doi: 10.1186/s13046-024-03095-8 (PMC11223299; doi:10.1186/s13046-024-03095-8)

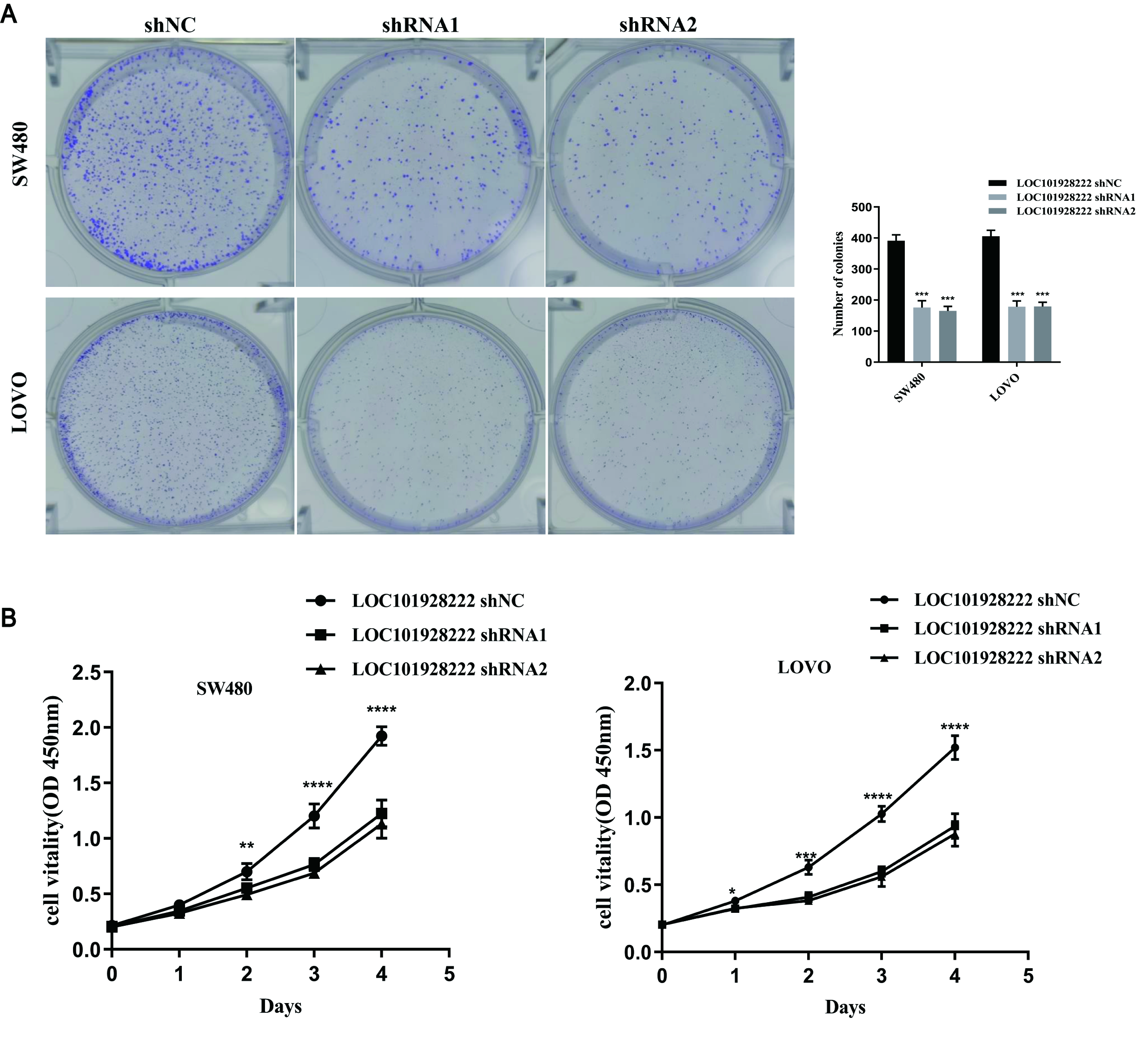

Supplement: Supplementary file 3 — Additional file 3: Figure S1. LOC10192822 promotes colorectal cancer (CRC) cell proliferation in vitro. (A) For LOC101928222 knockdown, colony forming assays assessed CRC cell proliferation. (B) For LOC10192888 knockdown, the Cell Counting Kit (CCK)-8 assay assessed CRC cell viability. * p< 0.05, **p < 0.01, ***p < 0.001, ****p < 0.0001. [file 13046_2024_3095_MOESM3_ESM.tif]

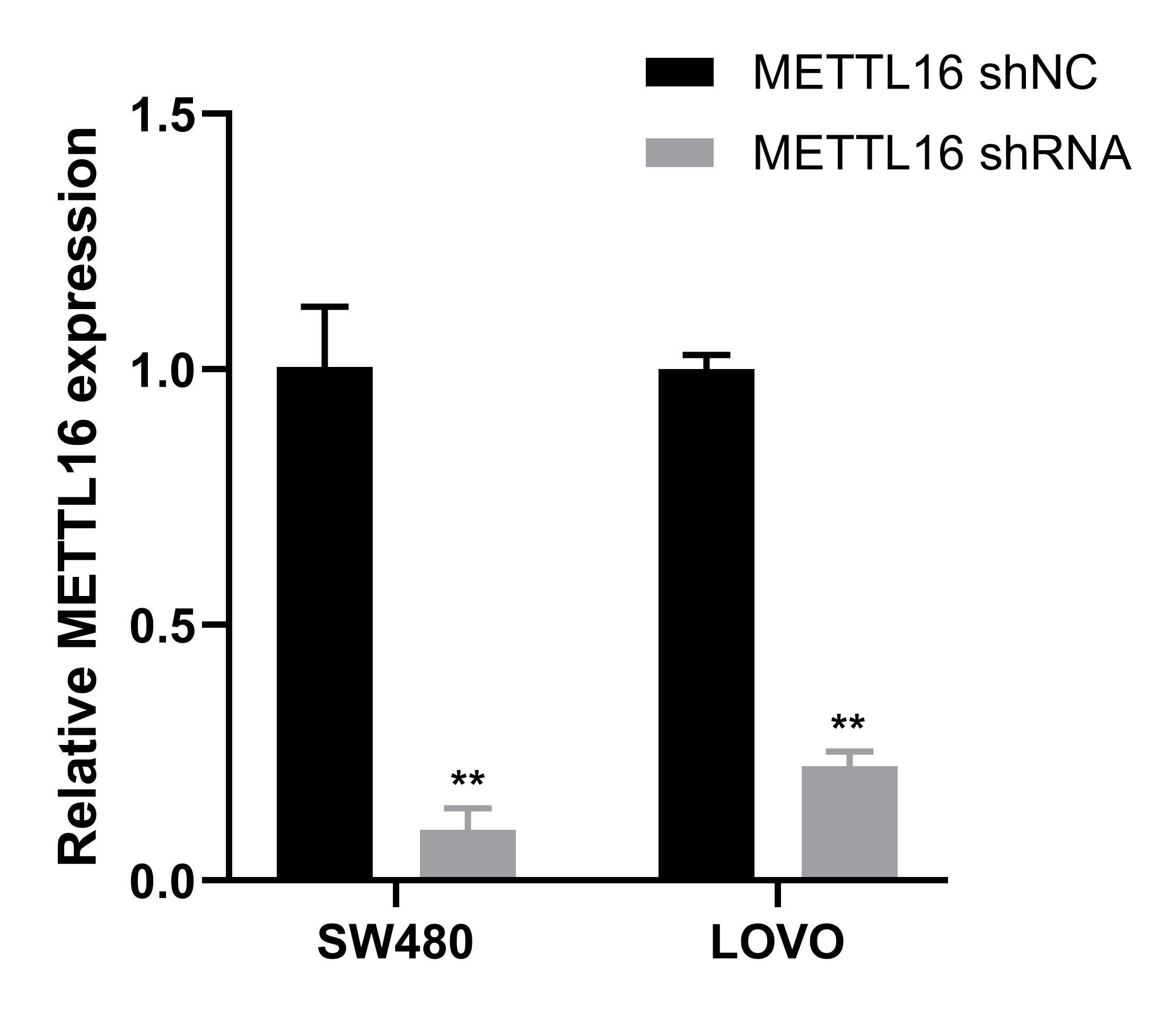

Supplement: Supplementary file 4 — Additional file 4: Figure S2. The interference efficiency of METTL16 were verified by qRT-PCR in SW480 and LOVO cells. [file 13046_2024_3095_MOESM4_ESM.tif]

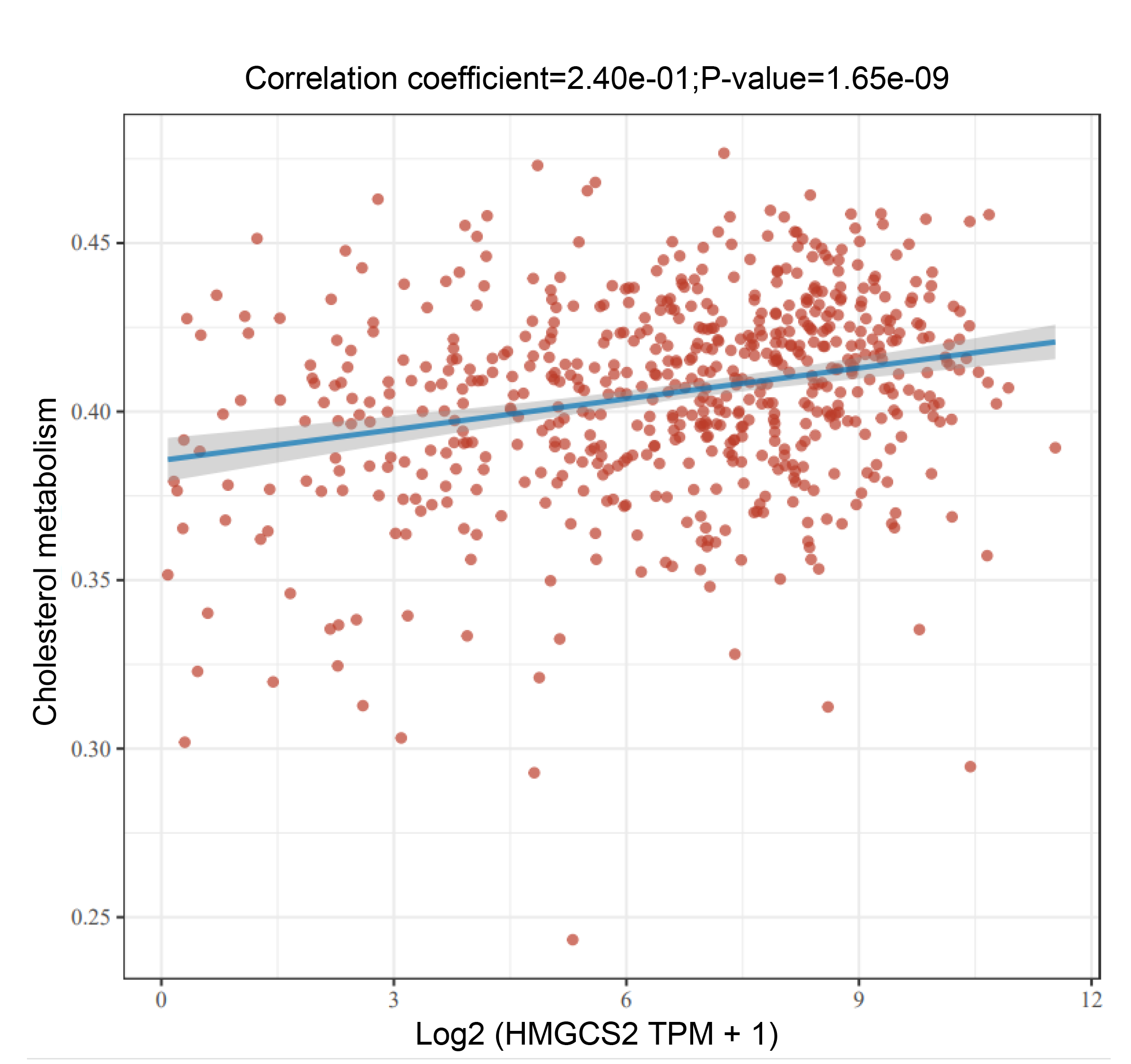

Supplement: Supplementary file 5 — Additional file 5: Figure S3. Bioinformatics analysis of the relationship between HMGCS2 and cholesterol metabolism. [file 13046_2024_3095_MOESM5_ESM.tif]
